# The effectiveness and cost-evaluation of manual therapy and physical therapy in patients with sub-acute and chronic non specific neck pain. Rationale and design of a Randomized Controlled Trial (RCT)

**DOI:** 10.1186/1471-2474-11-14

**Published:** 2010-01-24

**Authors:** Ruud Groeneweg, Hans Kropman, Huco Leopold, Luite van Assen, Jan Mulder, Maurits W van Tulder, Rob AB Oostendorp

**Affiliations:** 1 Radboud University Nijmegen Medical Centre, Scientific Institute for Quality of Health Care, P.O. Box 9101, 6500 HB Nijmegen, The Netherlands; 2 Practice for Manual Therapy, Filomeentje-erf 154, 2907 BC Capelle aan den IJssel, The Netherlands; 3 Practice for Manual Therapy, Bellestein 61 G, 6714 DP Ede, The Netherlands; 4 Practice for Manual Therapy, Den Bloeyenden Wijngaerdt 3c, 1183 JM Amstelveen, The Netherlands; 5 Practice for Physical Therapy and Manual Therapy, Wijnstraat 110, 3311 BX Dordrecht, The Netherlands; 6 Department of Health Sciences & EMGO Insitute for Health and Care Research, Faculty of Earth & Life Sciences, VU University Amsterdam; 7 Dutch Institute of Allied health Care, P.O. Box 1161, 3800 BD Amersfoort, The Netherlands

## Abstract

**Background:**

Manual Therapy applied to patients with non specific neck pain has been investigated several times. In the Netherlands, manual therapy as applied according to the Utrecht School of Manual Therapy (MTU) has not been the subject of a randomized controlled trial. MTU differs in diagnoses and treatment from other forms of manual therapy.

**Methods/Design:**

This is a single blind randomized controlled trial in patients with sub-acute and chronic non specific neck pain. Patients with neck complaints existing for two weeks (minimum) till one year (maximum) will participate in the trial. 180 participants will be recruited in thirteen primary health care centres in the Netherlands.

The experimental group will be treated with MTU during a six week period. The control group will be treated with physical therapy (standard care, mainly active exercise therapy), also for a period of six weeks.

Primary outcomes are Global Perceived Effect (GPE) and functional status (Neck Disability Index (NDI-DV)). Secondary outcomes are neck pain (Numeric Rating Scale (NRS)), Eurocol, costs and quality of life (SF36).

**Discussion:**

This paper presents details on the rationale of MTU, design, methods and operational aspects of the trial.

**Trial registration:**

ClinicalTrials.gov Identifier: NCT00713843

## Background

Neck pain is one of the most common complaints of the musculoskeletal system.

Approximately two thirds of the population will at some point in their life experience neck pain [1]. Prevalence rises with age in both sexes and is highest at ages between 50 and 59. In general women suffer from neck complaints twice as often as men.

Prevalence of neck complaints is between 10% and 15% [2]. In the Netherlands, the point prevalence in absolute figures, calculated in the year 2000, amounted to 594,000 males and 1,013,700 females registered with chronic neck complaints [3]. Systematic reviews showed a considerable heterogeneity in prevalence of neck complains. Fejer et al found a range of the one-month prevalence from 15.4% to 41% [4]. Hogg-Johnson found a range from 15.4% to 45.3% among adults, interfering with activities ranged from 7.5% to 41.5% [5]. Bot showed an incidence of 23.1 per 1000 person-years of neck symptoms in a Dutch national survey of general practice [6].

The total costs related to neck pain in the Netherlands added up to approximately 668 million Euros in 1996. Direct medical costs amounted to 160 million Euros (23% of the total costs related to neck complaints) [2]. Allied health care (such as occupational therapy and physical therapy) made up the largest proportion of the direct costs (84%) [2]. As such, neck pain forms a significant personal and economical problem.

Neck pain can be caused by traumata (specifically traffic accidents), infections, tumours, congenital defects and inflammations; however in many cases it is not possible to determine the underlying cause. In these cases neck pain will be regarded as 'non-specific neck pain'.

In case of acute neck pain the general practitioner (GP) will usually not take immediate action. Pain medication might be prescribed [7]. Research by Vos indicates that 51% of patients having neck pain are referred to physical therapy or manual therapy [7].

These figures may differ internationally because of different referral policy.

If the complaints are persistent (for six months or longer) it appears that the average discomfort perceived will remain fairly stable [8]. It is clinically and economically relevant to ensure that patients do not end up in this chronic phase.

### Level of evidence manual therapy

In 2004 Gross et al made a meta-analysis of Randomized Controlled Trials (RCTs) in which the effect of manipulations and mobilizations for mechanical neck complaints was investigated [9]. In cases of a single manipulation session or several sessions (3 to 11 weeks) of manipulations or mobilizations compared to a control group or when mobilizations were compared to other forms of therapy, moderate evidence was found that this gave no results in the short term for acute, sub-acute and chronic mechanical neck problems.

When manipulation and mobilization were compared to withholding treatment, the results showed a tendency towards a positive effect of manipulation and mobilization. Mobilisation and manipulation compared to placebo or control groups showed a nonsignificant result [9].

Their conclusions where that the evidence did not favour manipulation and/or mobilisation done alone or in combination with various other physical medicine agents; when compared to one other, neither was superior.

As for the costs of care, there was moderate evidence that manual therapy was less expensive than other forms of care in acute, sub-acute and chronic neck complaints with or without headache or cervical radicular signs [9].

In 2007, Vernon et al published a systematic review of applying manual therapy in cases of neck pain [10]. Little evidence was found about the application of manual therapy for acute neck pain (existing less than four weeks) [10]. There was moderate- to high-quality evidence that subjects with chronic neck pain show clinically important improvements from a course of spinal manipulation or mobilization, using intragroup changes [11]. The Task Force on Neck Pain stated that manual therapy or exercise therapy was not clearly superior to one other in either short- or long-term [12].

In some countries manual therapy has been included in the guidelines for treatment of neck pain [11].

In summary, reviews showed that manual therapy is an effective method (intragroup changes)(specially in sub acute and chronic neck pain) but compared to controls there is in general no clear evidence that one of them is superior (intergroup changes).

### Research in the Netherlands

In the Netherlands, the effectiveness and cost-effectiveness of treatments with manual therapy and physical therapy (PT) compared with counselling of the general practitioner (GP) for non-specific neck pain was investigated in a RCT [13].

MT consisted of mobilizing techniques as described by Van der El en Di Fabio [14,15]. Low-amplitude, high-velocity thrust techniques were not applied. After seven weeks, perceived recovery was 68% for MT, 51% for PT and 36% for GP treatment. MT scored statistically significantly better than the other interventions. PT scored better than GP, although this difference was non-significant. At one year follow-up there were no statistically significant differences between the groups for pain and impediment (global perceived effect after one year 72% MT, 63% PT and 56% GP) [16]. MT was significantly more cost-effective than treatment by the GP and physical therapy [17]. The total costs per patient, including absence from work and costs of health care utilisation, amounted to €447 for MT, €1297 for PT and €1379 for GP.

In later research by Pool et al manual therapy (MT) was compared with exercise therapy with behaviour graded activity (BGA) in people with neck complaints [18]. Pool found neither clinically relevant nor statistically significant differences. 89.4% in the BGA group and 86.5% in the MT group showed positive global perceived effect after 52 weeks.

So, although the systematic reviews did not clearly show an effect of manual therapy for neck pain, two Dutch studies found that manual therapy is more cost-effective than physiotherapy and GP counselling, and equally effective as an extensive exercise therapy with behaviour graded activity programme. However, in both Hoving's and Pool's studies other forms of manual therapy than MTU were used [13,19].

### Manual therapy according to the Utrecht School (MTU)

MTU is based on assessing the patient's individual preference of functioning by documenting and interpreting their natural asymmetry in anatomical form, posture and movements.

The normal asymmetry and variability of human form and movement function have been specified in research [20-26]. These studies showed that many movements are carried out asymmetrically. These asymmetrical forms can be related to the asymmetrical movement function.

In addition to the general diagnostics, MTU is characterized by specific diagnostics. By means of this specific manual-therapeutic analysis the individual preference of functioning model of the patient is drawn up through documentation and interpretation of the individual asymmetry in form, posture and movement [27,28]. Some explanations of the measurements and movements are: (preferred) hand folding; (preferred) arm folding; which eye is master eye; leg use in (preferred) kicking of a ball. The purpose here is to describe the optimal direction and position of movement axes for all joints according to this model.

When composing this model, firstly the individual characteristics (a number of preferred movements, a number of asymmetrical aspects of posture and form) are assessed. Documentation, notation and interpretation of these characteristics take place according to a protocol.

The objective of MTU is to optimise the positioning of movement axes in the joints. To achieve this, three-dimensional movements in the joints are executed repeatedly. To purpose fully position the movement axes the therapist should (repeatedly) perform passive joint movements with low velocity and high accuracy. In addition to examining the individual preference of movement, exploratory examination is carried out to recognise possible red flags and to determine the treatment indication.

Treatment is based on preferred movements found in the patient and the interpretation according to the protocol of these movements and not on the complaint of the patient. It is executed by applying passive articular movements in the spinal joints and the joints of the extremities. During this process physiological joint limitations are carefully observed; traction or high-velocity movements will not be applied, as may be the case in other forms of manual therapy [29].

The diagnostic examination of other forms of manual therapy focuses on joint function, stability, movement patterns, range of movement, and the severity of disorders [30]. To diagnose the patients complaints, palpation of passive accessory and passive intervertebral movements are used. The results yield information as to tenderness (pain), restricted intersegmental motion (stiffness), and spasm (muscle tension) [29-31].

In general, other manual therapies, as described by Veen et al, are directed primarily to the complaints of patients, particularly the main complaint [29,32].

### Trial Objectives

The primary objective is to compare the short-term effectiveness (7 weeks) and long-term effectiveness (52 weeks) of MTU with physical therapy in patients with sub-acute and chronic neck pain with regard to global perceived effect, functioning and pain.

The cost-effectiveness of manual therapy compared with physiotherapy will also be evaluated.

## Methods/Design

### Design and setting

This research is a single-blinded randomized controlled trial with cost-evaluation. A central research centre is set up in the Radboud University Nijmegen Medical Center, the Netherlands, housing a central investigator, an advisor, an independent physician, a statistician and a blinded research assistant entering data.

There are twelve locations where patients are treated. All of these are primary health care centres for manual and/or physical therapy. Each local centre will have a manual therapist, a physical therapist and a research assistant.

### Ethical approval

Ethical approval was obtained from the Medical Ethics committee CMO Arnhem-Nijmegen (NL21128.091.08).

### Inclusion and exclusion

Males and females aged between 18 and 70 years having neck pain for at least two weeks and with the last episode started at maximum of 52 weeks ago are eligible. Neck pain is the primary pain at the time of inclusion and must be provoked and reproduced as mechanical neck pain by movement or posture of the neck. Participants may have cervicogenic headache and radiation to the elbow. Exclusion criteria are: presence of red flags such as specific neck pain caused by cervical radiculopathy, entrapment neuropathy, myelopathy, unexplained fever, unexplained weight loss, nocturnal persistent pain, general malaise [33]; surgery of the cervical spine; pregnancy; whiplash trauma (in the past or recent, as cause of the complaint); physical conditions seriously impeding treatment (such as amputations, being wheelchair bound, illness); insufficient knowledge and command of the Dutch language for answering the questionnaires (to be judged by the research assistant); therapeutic treatment for neck pain in the previous three months such as physical therapy, manual therapy, osteopathy, chiropractics and acupuncture.

### Sample size

The sample size is based on one of the two primary outcome variables, Neck Disability Index (NDI) and Global Perceived Effect (GPE). The GPE is chosen, because this outcome variable needs the largest group of participants. Previous studies have shown that the effect of manual therapy on GPE is 68.3% [13] and 70.1% [18].

A 20% difference on the GPE scale is considered clinically relevant. Based on α = .05 and an 80% power (β = 0.2) 76 participants per intervention group are required.

With regard to prospective drop outs (15%), 90 participants per group will be recruited in this trial. A similar sample size was used in previous research [19,34,35].

### Interventions

#### MTU (experimental intervention)

**Figure 1:**
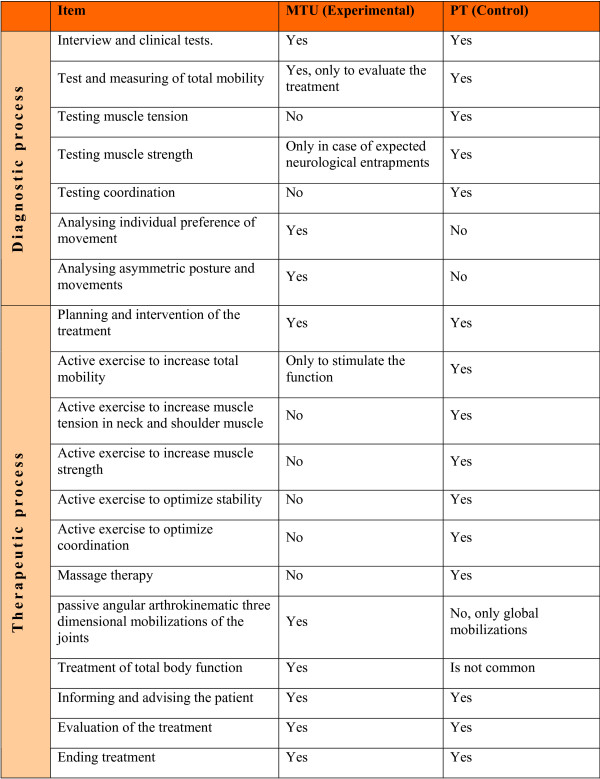
**Shows the differences of the intervention between the experimental group MTU (Manual therapy Utrecht) and the control group PT (physical therapy)**.

During the first consultation the manual therapist enquires about the complaints of the patient. The manual therapist conducts a number of measurements according to protocol, thus registering the natural asymmetry in form, posture and movement (see figure 1).

By means of an interpretation according to the protocol the measurements are translated into preferred movements in the patient's joints. During treatment these preferred movements are executed by the manual therapist in the patient's joints. The treatment techniques used by the manual therapist are very gentle mobilizations, without high velocity thrust techniques and are in general painless. In MTU it is common to give advice and recommend exercise.

A treatment session lasts between 30 and 60 minutes. In this trial treatment is repeated after one or two weeks. The maximum number of sessions is six.

The manual therapist has a minimum of five years of working experience.

#### Physical therapy (comparison intervention)

At the first appointment the physical therapist enquires about the complaints. The physical therapist conducts a complaint related function examination, after which treatment goals are determined. Treatment can consist of active exercises, manual traction or stretching and massage. The aims of exercises are improvement of strength, mobility and movement coordination. Specific manual mobilization techniques, known as manual-therapeutic techniques, are not a part of physiotherapeutic treatment. Treatment sessions take place no more than twice a week with a maximum of nine sessions; session duration is approximately 30 minutes. In each session the physical therapist will spend a minimum of twenty minutes on active exercise therapy combined with instruction.

To prevent overlap with MTU, physical therapists are selected who are not (also) trained as manual therapists or have started this training.

The physical therapist has at least five years of working experience.

Figure 1 contains the description of the experimental and control intervention.

#### Co-interventions

During the intervention period of the trial, participants will not receive treatment other than the ones allocated. Patients are free to use medication prescribed either by a physician or of their own choice. Participants are allowed to withdraw from the treatment at any time. Continuation of the treatment and co-interventions are registered.

### Outcome

In the choice of primary and secondary outcome the ICF (International Classification of Functioning, Disability and Health) components have been taken into account. These cover the following categories: bodily functions, anatomical properties; activities and participation; external factors; personal factors [36].

#### Primary outcome

Global Perceived Effect (GPE) measures overall improvement or worsening. Measuring of GPE will be done by scoring a 7-point ordinal scale (ranging from much worse to complete recovery) [37,38]. The GPE measures patient subjective global improvement and has a high face validity [39]. In routine clinical practice it is important, since it would not make sense to classify a patient as improved or deteriorated against the patient's own personal assessment [39,40]. Functioning is the second primary outcome. The Neck Disability Index Dutch Version (NDI-DV) is a questionnaire containing 10 items. All items are related to daily functioning and functions. The maximum score is 50; the higher the score the more limitations. Validity and reliability of the NDI are good [41,42] and so is the responsivity [42-44].

#### Secondary outcomes

To assess neck pain intensity the Numeric Rating Scale (NRS) will be used. This scale (11 points) measures the pain intensity experienced by the patient in the previous week. Dalton et al argued for standardization of pain measuring by means of the 11-point Numeric Rating Scale (NRS) [45]. The Visual Analogue Scale (VAS) and the NRS are the most cited pain measures, largely because they are simple to use. The NRS is a sensitive instrument, comparable with the VAS [46,47] or more sensitive than the VAS [48]. The NRS has been chosen on the basis of administrative aspects rather than for statistical power. It has been proved to be more comprehensible for patients [45-47,49,50]. Also the NRS is valid for verbal patient questioning [51].

The general health questionnaire (SF36) will be used to put together a detailed health profile on the basis of scores on eight health dimensions as well as a sum score on both physical and mental health [52].

The EuroQol5D is a standardized, non-disease-specific instrument for describing and valuing health states. It has the additional possibility of converting the descriptive data into values for economic (cost-effectiveness) analysis by linking patients' health state descriptions to empirical valuations of health states obtained from the general population [53]. EuroQol5D is simple to use, valid, responsive to change and reliable instrument for group comparisons [54-56]. The EuroQol5D is a two-part instrument. Part one records self-reported problems on each of five 'domains': mobility, self-care, usual activities, pain/discomfort and anxiety/depression. Each domain is divided into three levels of severity corresponding to no problem, some problem and extreme problem. Part two of the questionnaire records the subject's self-assessed VAS rating of health.

Participants are to fill in questions about (partial) disablement/return to work (if applicable), use of analgesic (types of and quantities), medical consultation during treatment and follow/up, costs and side effects [57,58].

The patient will fill in the questionnaires at baseline, 3, 7, 13, 26, 39 and 52 weeks after treatment has started (see table 1).

**Table 1:** Timing of measurements

measurements	Baseline T0	T3	T7	T13	T26	T39	T52
In- and exclusion	X						

Demographic data	X						

GPE		X	X	X	X		X

NDI-DV	X	X	X	X	X		X

NRS pain	X	X	X	X	X		X

SF-36	X		X				X

Side effect		X	X	X			

EuroQol5D		X	X	X	X	X	X

Costs		X	X	X	X	X	X

GPE = Global Perceived Effect (7-points scale); NDI-DV = Dutch version Neck Disability Index; NRS = Numeric Rating Scale for pain; SF36 = short form -36 quality of life questionnaire

### Procedure

#### Recruitment

**Figure 2:**
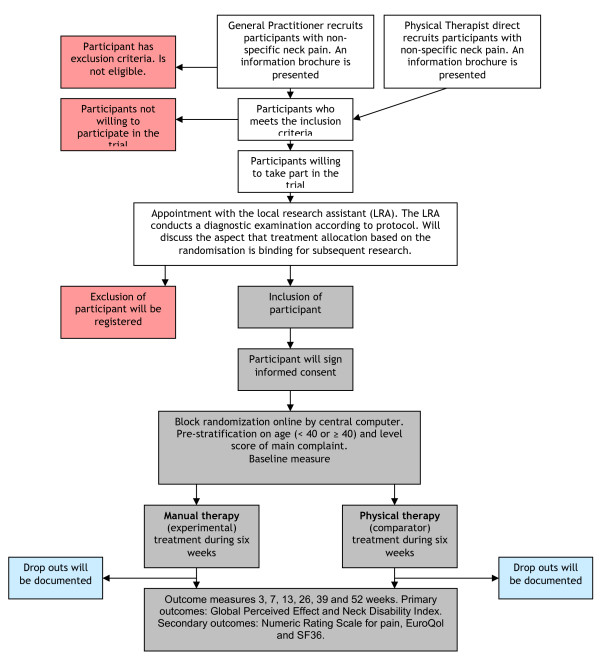
**NECKproject flow chart**.

The GP and therapists will send in patients suitable to include. See the flow chart in figure 2. The GP and therapists inform the patient about the aim of the study and present an information brochure. If the patient is willing to participate in the study, the GP or therapist contacts the local research assistant (LRA).

The LRA schedules an appointment with the patient. The patient will be informed again by the LRA about the contents and objectives of the study. After this, the LRA conducts a diagnostic examination according to protocol. Training and an instructional DVD is provided by the NECKprojectgroup to the LRA to ensure a correct intake procedure.

The purpose of the intake procedure is to assess inclusion and exclusion criteria. Should the patient be eligible, he/she is informed about how to fill in the questionnaires either digitally or on paper. After signing the informed consent, randomisation takes place. The patient receives an envelope with the name and address of the (local) therapist that will provide the treatment. The patient makes an appointment with the therapist in attendance and starts treatment. The LRA has no further contact with the patient in relation to this trial.

All questionnaires are sent either digitally or by mail to the central blinded investigator.

#### Randomisation, blinding and allocation

Block randomisation will take place through the computer after pre-stratification on the basis of prognostic aspects for the complaints: level score of the main complaint (scale 10: <7 or ≥ 7) and age (< 40 or ≥ 40 years) [59,60].

Four groups are formed which are randomized for both intervention groups. The randomisation scheme will be generated by the central computer. The LRA is blinded for the randomisation.

Treatment with physical therapy and MTU will take place at different locations. The investigator in charge of the database has no access to the key of the combination patient data - research number - treatment allocation. In the case of missing data the computer system will automatically generate an email to the participant directly or to the secretary of the NECKprojectgroup. The computer or secretary will send the patient a standard reminder. None of the therapists know the patient number(s).

The data from the questionnaires filled in digitally on http://www.neckproject.nl are sent directly to the database of the central researcher. The researcher is blinded from patients' data and type of intervention. The questionnaires filled in by hand are sent to a central freepost address. These lists are entered into the database by a secretary. Loss of data should be prevented by actively reminding the patient by email, mail or telephone.

Patients who fill in the forms digitally will automatically receive a reminder by email. Patients who fill in the lists by hand will receive them through the post. The secretary will attend to this.

Each treatment sessions will be registered by using standard forms filled in by the therapist. By doing spot checks the information received about the content of the treatment session will be inspected by the researcher from the UMC St Radboud.

Visitations will take place to ensure the therapists in attendance are keeping to the research protocols.

#### Side effects

All side-effects, both reported spontaneously by patients and noticed by the therapist, are registered. Any serious side-effects are registered and reported to the Medical Ethic Commission in charge, according to the rules of this organisation.

### Statistics

#### Descriptive statistics

Demographic data (gender, average age, disability/fitness, duration of complaints, headache of cervical origin, use of medication and other items) will be presented.

Outcomes will be presented per group (baseline, MTU, physical therapy) in mean, standard deviation and 95% confidence interval.

#### Multi-variate analysis

An ANOVA will be used to analyze differences between conditions per person (within subject) and per group (between groups).

Descriptive statistics will be applied to make a comparison between the baseline data of the manual therapy and the physical therapy groups and to assess whether the randomisation has been successful. Group differences between the interventions with a 95% confidence interval will be calculated for all outcome measures.

The statistical analyses will be carried out according to the intention-to-treat principle, whereby the patients are analysed in the intervention group in which they were originally placed. The differences between groups will be tested by ANOVA/mixed model (continuously) and by the Chi-Square test for dichotomous variables.

Multi-variate regression analysis will be used to test the influence of the baseline variables on the outcomes.

For the primary outcome measure Global Perceived Effect (GPE) a selection can be made between responders (positive effect) and non-responders (no or negative effect) to the treatment received.

The data will be processed by SPSS/SAS statistical software.

## Discussion

In working with local centres, locally approaching the GPs by the manual therapists and physical therapists, we expect to be able to have the required number of participants minimally within one year after the start. Trial recruitment started in September 2008.

## Competing interests

The authors declare that they have no competing interests.

## Authors' contributions

RG, HK, HL, LA, JM, MT and RO were responsible for the design of the trial. All authors read and approved the final manuscript.

## Pre-publication history

The pre-publication history for this paper can be accessed here:

http://www.biomedcentral.com/1471-2474/11/14/prepub

## References

[B1] Binder A Neck pain Clin Evid 2006 15 1654 1675 16973064

[B2] Borghouts JA Koes BW Vondeling H Bouter LM Cost-of-illness of neck pain in The Netherlands in 1996 Pain 1999 80 3 629 636 10.1016/S0304-3959(98)00268-1 10342424

[B3] Nationaal Kompas Volksgezondheid versie 3.10 2007 Bilthoven RIVM

[B4] Fejer R Kyvik KO Hartvigsen J The prevalence of neck pain in the world population: a systematic critical review of the literature Eur Spine J 2006 15 834 848 10.1007/s00586-004-0864-4 15999284 PMC3489448

[B5] Hogg-Johnson S Velde G van der Carroll LJ Holm LW Cassidy JD Guzman JJ Côté P Haldeman S Ammendolia C Carragee E Hurwitz E Nordin M Peloso P The Burden and Determinants of Neck Pain in the General Population. Results of the Bone and Joint Decade 2000-2010 Task Force on Neck Pain and Its Associated Disorders Spine 2008 33 4S S39 S51 10.1097/BRS.0b013e31816454c8 18204398

[B6] Bot SDM Waal JM van der Terwee CB Windt DAWM van der Schellevis FG Bouter LM Dekker J Incidence and prevalence of complaints of the neck and upper extremity in general practice Ann Rheum Dis 2005 64 118 123 10.1136/ard.2003.019349 15608309 PMC1755209

[B7] Vos C Verhagen A Passchier J Koes B Management of acute neck pain in general practice: a prospective study Br J Gen Pract 2007 57 534 23 28 17244420 PMC2032696

[B8] Borghouts JA Koes BW Bouter LM The clinical course and prognostic factors of non-specific neck pain: a systematic review Pain 1998 77 1 1 13 10.1016/S0304-3959(98)00058-X 9755013

[B9] Gross A Hoving J Haines T Goldsmith C Kay T Aker P Bronfort G Manipulation and mobilisation for mechanical neck disorders Cochrane Database Syst Rev 2004 1 CD004249 14974063 10.1002/14651858.CD004249.pub2

[B10] Vernon H Humphreys BK Manual therapy for neck pain: an overview of randomized clinical trials and systematic reviews Eura Medicophys 2007 43 1 91 118 17369783

[B11] Vernon H Humphreys K Hagino C Chronic mechanical neck pain in adults treated by manual therapy: A systematic review of change scores in randomized clinical trials J Manipulative Physiol Ther 2007 30 215 227 10.1016/j.jmpt.2007.01.014 17416276

[B12] Hurwitz EL Carragee EJMD Velde G van der Carroll LJ Nordin M Guzman Treatment of Neck Pain: Noninvasive Interventions. Results of the Bone and Joint Decade 2000-2010 Task Force on Neck Pain and Its Associated Disorders Spine 2008 33 4S 123 152 10.1097/BRS.0b013e3181644b1d 18204386

[B13] Hoving JL Koes BW de Vet HC Windt DA van der Assendelft WJ van Mameren H Deville WL Pool JJ Scholten RJ Bouter LM Manual therapy, physical therapy, or continued care by a general practitioner for patients with neck pain. A randomized, controlled trial Ann Intern Med 2002 136 10 713 722 12020139 10.7326/0003-4819-136-10-200205210-00006

[B14] Avd EL Lunacek P Wagemaker A Manuele Therapie: wervelkolom behandeling [Manual Therapy: Treatment of the Spine] 1993 2 Rotterdam: Manuwel

[B15] Di Fabio RP Manipulation of the cervical spine: risks and benefits Phys Ther 1999 79 1 50 65 9920191

[B16] Hoving J de Vet H Koes B Mameren H Deville W Windt D van der Assendelft W Pool J Scholten R Korthals-de Bos I Bouter LM Manual therapy, physical therapy, or continued care by the general practitioner for patients with neck pain: long-term results from a pragmatic randomized clinical trial Clin J Pain 2006 22 4 370 377 10.1097/01.ajp.0000180185.79382.3f 16691091

[B17] Korthals-de Bos IB Hoving JL van Tulder MW Rutten-van Molken MP Ader HJ de Vet HC Koes BW Vondeling H Bouter LM Cost effectiveness of physiotherapy, manual therapy, and general practitioner care for neck pain: economic evaluation alongside a randomised controlled trial Bmj 2003 326 7395 911 10.1136/bmj.326.7395.911 12714472 PMC153837

[B18] Pool J Ostelo R Knol D Vlaeymen J Bouter L de Vet R Pool JJM Is a behavioural graded activity programme more effective than manual therapy in patients with sub-acute neck pain? Results of a pragmatic randomized clinical trial Neck pain: "a pain in the neck?". A study of therapeutic modalities and clinimetrics 2007 Amsterdam: Proefschrift Vrij Universiteit Amsterdam

[B19] Pool JJ Ostelo RW Koke AJ Bouter LM de Vet HC Comparison of the effectiveness of a behavioural graded activity program and manual therapy in patients with sub-acute neck pain: design of a randomized clinical trial Man Ther 2006 11 4 297 305 10.1016/j.math.2005.07.006 16380288

[B20] Boulay C Three-dimensional study of pelvic asymmetry on anatomic specimens and its clinical perspectives Journal of Anatomy 2006 208 21 33 10.1111/j.1469-7580.2006.00513.x 16420376 PMC2100175

[B21] Dumas JL Salama J Dreyfus P Thoreux P Goldlust D Chevrel JP Magnetic resonance angiographic analysis of atlanto-axial rotation: anatomic bases of compression of the vertebral arteries Surg Radiol Anat 1996 18 4 303 313 10.1007/BF01627609 8983110

[B22] Dumas JL Thoreux P Attali P Goldlust D Chevrel JP Three-dimensional CT analysis of atlantoaxial rotation: results in the normal subject Surg Radiol Anat 1994 16 2 199 204 10.1007/BF01627595 7940085

[B23] Gottlieb MS Absence of symmetry in superior articular facets on the first cervical vertebra in humans: implications for diagnosis and treatment J Manipulative Physiol Ther 1994 17 5 314 320 7930965

[B24] Kuhtz-Buschbeck JP Brockmann K Gilster R Koch A Stolze H Asymmetry of arm-swing not related to handedness Gait Posture 2007 27 3 447 54 10.1016/j.gaitpost.2007.05.011 17616462

[B25] Penning L Normale bewegingen van de hals- en lendenwervelkolom 1998 Utrecht: Lemma

[B26] Ross JK Bereznick DE McGill SM Atlas-axis facet asymmetry. Implications in manual palpation Spine 1999 24 12 1203 1209 10.1097/00007632-199906150-00006 10382246

[B27] Bijl Gvd Het individuele functiemodel in de manuele therapie 1986 Lochem: De Tijdstroom

[B28] Cock Jd Begrippen van manuele therapie, Systeem Van der Bijl 1996 Utrecht: De Tijdstroom

[B29] Veen Evd Vet Hd Pool JJ Schuller W Zoete Ad Bouter L Variance in manual treatment of nonspecific low back pain between orthomanual physicians, manual therapists, and chiropractors J Manipulative Physiol Ther 2005 28 2 108 116 10.1016/j.jmpt.2005.01.008 15800510

[B30] Baumgarten K Hoppenbrouwers G Wurff P Van der Oostendorp R Heerkens Y Functieprofiel Manueel Therapeut, versie 1.0 [Functional profile manual therapy] 1996 Amersfoort: Nederlands Paramedisch Instituut

[B31] Gross AR Aker PD Quartly C Manual therapy in the treatment of neck pain Rheum Dis Clin North Am 1996 22 3 579 598 10.1016/S0889-857X(05)70289-1 8844915

[B32] Aalberse R Esch M van de Groeneweg R Van Helvoirt J Oostendorp R Peeters G Landelijk functie opleidingsprofiel Manuele Therapie [Educational profile manual therapy] 2001 Amersfoort: Nederlands Paramedisch Instituut

[B33] Binder AI Cervical spondylosis and neck pain Bmj 2007 334 7592 527 531 10.1136/bmj.39127.608299.80 17347239 PMC1819511

[B34] Luijsterburg PA Verhagen AP Ostelo RW Hoogen HJ van den Peul WC Avezaat CJ Koes BW Conservative treatment in patients with an acute lumbosacral radicular syndrome: design of a randomised clinical trial [ISRCTN68857256] BMC Musculoskelet Disord 2004 5 1 39 10.1186/1471-2474-5-39 15535882 PMC534096

[B35] Vonk F Verhagen AP Geilen M Vos CJ Koes BW Effectiveness of behavioural graded activity compared with physiotherapy treatment in chronic neck pain: design of a randomised clinical trial [ISRCTN88733332] BMC Musculoskelet Disord 2004 5 1 34 10.1186/1471-2474-5-34 15469609 PMC526281

[B36] WHO FIC Collaborating Centre in the Netherlands RIVM Nederlandse vertaling van de WHO-publicatie: International Classification of Functioning, Disability and Health: ICF, Geneva 2001 2002 1 Houten: Bohn Stafleu Van Loghum

[B37] Beurskens AJ de Vet HC Koke AJ Responsiveness of functional status in low back pain: a comparison of different instruments Pain 1996 65 1 71 76 10.1016/0304-3959(95)00149-2 8826492

[B38] Feinstein A (ed) Clinimetrics 1987 New Haven and London: Yale University Press

[B39] Roer Nvd Ostelo R Bekkering G van Tulder M de Vet H Minimal clinically important change for pain intensity, functional status, and general health status in patients with nonspecific low back pain Spine 2006 31 5 578 582 10.1097/01.brs.0000201293.57439.47 16508555

[B40] Kovacs FM Abraira V Royuela A Corcoll J Alegre L Cano A Muriel A Zamora J del Real MT Gestoso M Mufraggi N Minimal clinically important change for pain intensity and disability in patients with nonspecific low back pain Spine 2007 32 25 2915 2920 10.1097/BRS.0b013e31815b75ae 18246018

[B41] Hains F Waalen J Mior S Psychometric properties of the neck disability index J Manipulative Physiol Ther 1998 21 2 75 80 9502061

[B42] Vernon H The Neck Disability Index: state-of-the-art, 1991-2008 J Manipulative Physiol Ther 2008 31 7 491 502 10.1016/j.jmpt.2008.08.006 18803999

[B43] Pool JJ Ostelo RW Hoving JL Bouter LM de Vet HC Minimal clinically important change of the Neck Disability Index and the Numerical Rating Scale for patients with neck pain Spine 2007 32 26 3047 3051 10.1097/BRS.0b013e31815cf75b 18091500

[B44] Vos CJ Verhagen AP Koes BW Reliability and responsiveness of the Dutch version of the Neck Disability Index in patients with acute neck pain in general practice Eur Spine J 2006 15 11 1729 1736 10.1007/s00586-006-0119-7 16670840

[B45] Dalton JA McNaull F A call for standardizing the clinical rating of pain intensity using a 0 to 10 rating scale Cancer Nurs 1998 21 1 46 49 10.1097/00002820-199802000-00006 9494230

[B46] Williamson A Hoggart B Pain: a review of three commonly used pain rating scales J Clin Nurs 2005 14 7 798 804 10.1111/j.1365-2702.2005.01121.x 16000093

[B47] Breivik EK Bjornsson GA Skovlund E A comparison of pain rating scales by sampling from clinical trial data Clin J Pain 2000 16 1 22 28 10.1097/00002508-200003000-00005 10741815

[B48] Grotle M Brox JI Vollestad NK Concurrent comparison of responsiveness in pain and functional status measurements used for patients with low back pain Spine 2004 29 21 E492 501 10.1097/01.brs.0000143664.02702.0b 15507789

[B49] Herr KA Spratt K Mobily PR Richardson G Pain intensity assessment in older adults: use of experimental pain to compare psychometric properties and usability of selected pain scales with younger adults Clin J Pain 2004 20 4 207 219 10.1097/00002508-200407000-00002 15218405

[B50] Kim EJ Buschmann MT Reliability and validity of the Faces Pain Scale with older adults Int J Nurs Stud 2006 43 4 447 456 10.1016/j.ijnurstu.2006.01.001 16510146

[B51] Paice JA Cohen FL Validity of a verbally administered numeric rating scale to measure cancer pain intensity Cancer Nurs 1997 20 2 88 93 10.1097/00002820-199704000-00002 9145556

[B52] Aaronson NK Muller M Cohen PD Essink-Bot ML Fekkes M Sanderman R Sprangers MA te Velde A Verrips E Translation, validation, and norming of the Dutch language version of the SF-36 Health Survey in community and chronic disease populations J Clin Epidemiol 1998 51 11 1055 1068 10.1016/S0895-4356(98)00097-3 9817123

[B53] Brooks R EuroQol: the current state of play Health Policy 1996 37 1 53 72 10.1016/0168-8510(96)00822-6 10158943

[B54] Hurst NP Kind P Ruta D Hunter M Stubbings A Measuring health-related quality of life in rheumatoid arthritis: validity, responsiveness and reliability of EuroQol (EQ-5D) Br J Rheumatol 1997 36 5 551 559 10.1093/rheumatology/36.5.551 9189057

[B55] Agt HMv Essink-Bot ML Krabbe PF Bonsel GJ Test-retest reliability of health state valuations collected with the EuroQol questionnaire Soc Sci Med 1994 39 11 1537 1544 10.1016/0277-9536(94)90005-1 7817218

[B56] Essink-Bot ML Krabbe PF Bonsel GJ Aaronson NK An empirical comparison of four generic health status measures. The Nottingham Health Profile, the Medical Outcomes Study 36-item Short-Form Health Survey, the COOP/WONCA charts, and the EuroQol instrument Med Care 1997 35 5 522 537 10.1097/00005650-199705000-00008 9140339

[B57] Hurwitz EL Morgenstern H Vassilaki M Chiang LM Adverse reactions to chiropractic treatment and their effects on satisfaction and clinical outcomes among patients enrolled in the UCLA Neck Pain Study J Manipulative Physiol Ther 2004 27 1 16 25 10.1016/j.jmpt.2003.11.002 14739870

[B58] Rubinstein SM Leboeuf-Yde C Knol DL de Koekkoek TE Pfeifle CE van Tulder MW The benefits outweigh the risks for patients undergoing chiropractic care for neck pain: a prospective, multicenter, cohort study J Manipulative Physiol Ther 2007 30 6 408 418 10.1016/j.jmpt.2007.04.013 17693331

[B59] Hoving JL de Vet HC Twisk JW Deville WL Windt D van der Koes BW Bouter LM Prognostic factors for neck pain in general practice Pain 2004 110 3 639 645 10.1016/j.pain.2004.05.002 15288404

[B60] Koes B Hoving JL The value of the randomized clinical trial in the field of physiotherapy Manual Therapy 1998 3 4 179 186 10.1016/S1356-689X(98)80046-5

